# P-615. Encapsulation of Disease-Causing and Commensal Streptococcus Mitis and Other Viridans Streptococci

**DOI:** 10.1093/ofid/ofaf695.828

**Published:** 2026-01-11

**Authors:** Matthias Müsken, M John Hicks, Lesley McGee, Bernard Beall, Daniel M Musher

**Affiliations:** 3Helmholtz Centre for Infection Research, Braunschweig, Thuringen, Germany; Baylor College of Medicine, Houston, Texas; Centers for Disease Control and Prevention, Atlanta, Georgia; CDC, Atlanta, Georgia; Michael E. DeBakey VA Medical Center / Baylor College of Medicine, Houston, Texas

## Abstract

**Background:**

Viridans streptococci, specifically *Streptococcus mitis, S. infantis* and *S. oralis*, have been shown to cause bacteremia in immunocompromised hosts and, more recently, to be important causes of community-acquired pneumonia. These mitis group non-pneumococcal streptococci (MGNPS) often have capsular (*cps*) operons resembling those in pneumococci, and some may express *cps*-generated polysaccharides that antigenically cross-react with pneumococcal serotypes but, to date, a series of MGNPS isolates has not been studied by electron microscopy (EM) for the presence of a capsule.

**Figure:**
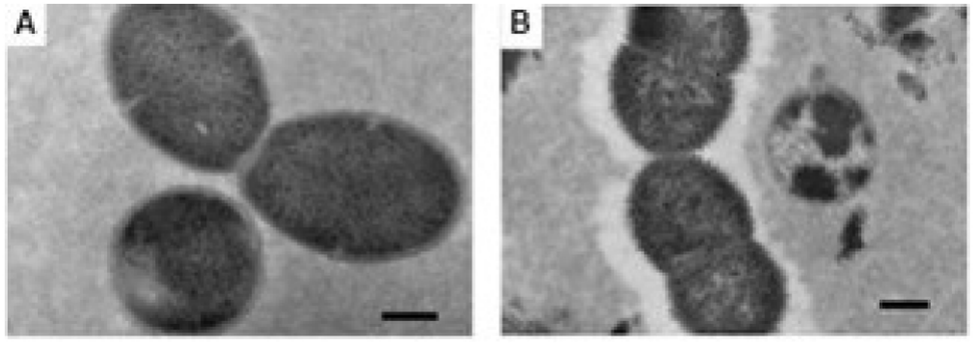
Transmission electron microscopy images after standard uranyl acetate fixation A. Streptococcus mitis showing no capsule; and B. S. pneumoniae serotype 5, showing clear evidence of a capsule. Scale bar 200 nm

**Figure:**
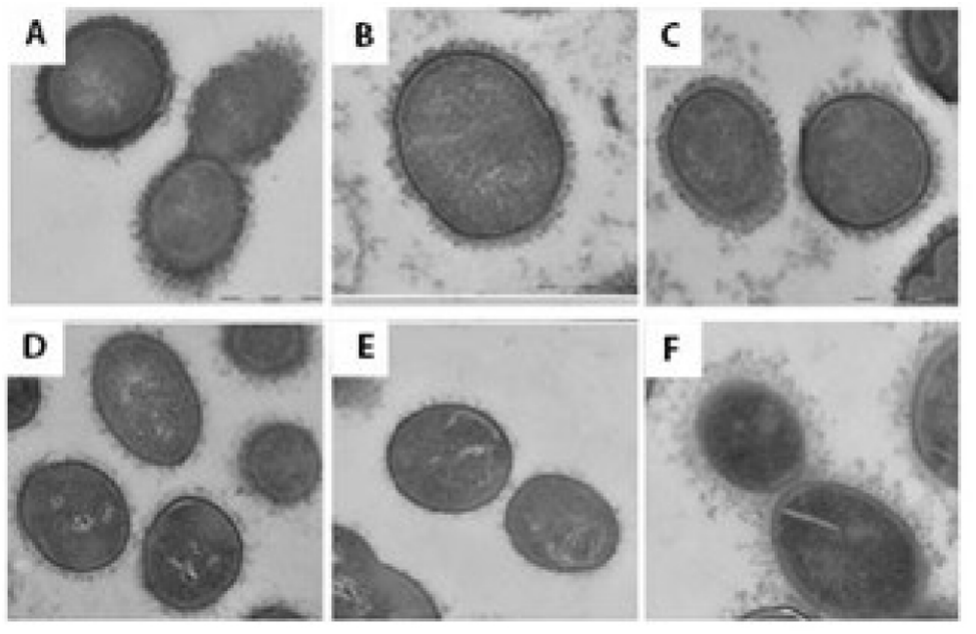
Transmission electron microscopy images after ruthenium red/lysine acetate fixation A. S. pneumoniae, type 15C, sputum isolate, patient with pneumonia. B. S. mitis, sputum isolate, patient with pneumonia. C. S. mitis commensal isolate from the mouth of a healthy adult. D. S. oralis, sputum isolate, patient with pneumonia; E. S. oralis, commensal isolate from the mouth of a healthy adult. F. S. mitis, commensal isolate with thick capsule, predicted as type 5 through the CDC bioformatics pipeline.

**Methods:**

We studied 21 MGNPS; 11 were isolated from sputum and determined to have caused pneumonia, 3 were isolated from blood, and 7 were commensal isolates cultured from the oral cavity of healthy adults. Isolates were fixed with two different protocols and examined by transmission EM.

**Results:**

EM after standard fixation and staining with uranyl acetate did not show capsules in MGNPS but clearly showed capsules in pneumococci (Figure 1A and 1B). In contrast, all MGNPS isolates studied after fixation with ruthenium red and lysine acetate were shown to be encapsulated. *S. pneumoniae* had the densest encapsulation (Fig 2A) followed by *S. mitis* (Fig 2B, 2C), *S. infantis* (not shown) and *S. oralis* (Fig 2D, 2E); in these MGNPS capsular material was more thinly spread over the bacterial surfaces. Within a species, there appeared to be no difference in capsules between disease-causing and commensal MGNPS strains. One *S. mitis* that identified as pneumococcus serotype 5 via the CDC bioinformatics platform had a thick capsule (Fig 1F) although it was not as dense as that seen on *S. pneumoniae*.

**Conclusion:**

This is the first EM demonstration of capsules on a randomly selected series of viridans streptococci. Importantly, within a species, there was no apparent difference in the capsules on disease-causing vs. commensal isolates. MGNPS are implicated as a cause in about 20% of cases of community-acquired pneumonia. We hypothesize that aspiration of a sufficiently large inoculum of relatively avirulent organisms in a person who has reduced clearance mechanisms or poorly functioning PMNs causes pneumonia. The precise role of capsule in the pathogenesis of pneumonia due to MGNPS remains to be elucidated.

**Disclosures:**

All Authors: No reported disclosures

